# Near Peer POCUS Education Evaluation 

**DOI:** 10.24908/pocus.v7i1.15019

**Published:** 2022-04-21

**Authors:** Cassidy Miller, Louisa Weindruch, John Gibson

**Affiliations:** 1 Texas College of Osteopathic Medicine Texas USA

**Keywords:** POCUS, near peer ultrasound, ultrasound, ultrasound education, near peer education, point-of-care ultrasound, point-of-care ultrasound education

## Abstract

**Objective**: At Texas College of Osteopathic Medicine (TCOM), point of care ultrasound (POCUS) is taught to medical students in conjunction with trained medical student teaching assistants (TAs). The purpose of our study is to evaluate the effectiveness of near peer teaching in the setting of ultrasound education. We hypothesized that this would be the preferred learning technique among TCOM students and TAs. **Methods**: To evaluate our hypotheses about the value of near peer instruction, we created two comprehensive surveys for students to share their experiences with the ultrasound program. One survey was for general students and the other survey was for students designated as TAs. The surveys were sent via email to second and third-year medical students. **Results**: **General Student Population Survey Results**: Of the 63 students who took the survey, 90.4% agreed that ultrasound is an integral part of medical education, 79.4% of students either agreed or strongly agreed that ultrasound improves their understanding of systems-based course material, 53.9% of students prefer near peer techniques over other teaching methods, while only 38.7% of students would prefer faculty-led sessions. 73% of students agreed that their ultrasound skills have improved with peer-led sessions, 71.4% of students agreed that peer-led sessions have made them want to pursue additional ultrasound training, and 96.8% of students report that they are very likely or somewhat likely to use POCUS in their future practice. **Ultrasound Teaching Assistant Survey Results**: Nineteen TAs responded to the survey, of which 78.9% assisted with more than 4 teaching sessions, 84.2% attended more than 4 TA training sessions, 94.7% reported spending additional time practicing ultrasound outside of TA activities each week, 100% agreed or strongly agreed that being an ultrasound TA has helped their medical education, and 78.9% either agreed or strongly agreed that they feel competent in their ultrasound skills. Among TAs, 78.9% preferred near peer techniques over other teaching methods, 100% agreed or strongly agreed that being a TA has helped develop their ultrasound skills, and 100% were likely or very likely to use POCUS in their future practice. **Conclusions**: Based on the results of our surveys, we were able to conclude that near peer teaching is the preferred learning method among students at our institution, and TCOM students found ultrasound to be a beneficial adjunct to systems courses in medical school education.

## Introduction

Point of care ultrasound (POCUS) is a diagnostic tool that is growing in both use and accessibility. Clinicians are no longer weighed down by a bulky machine when pocket ultrasound devices that connect to your phone or tablet are available. Due to its increased ease, POCUS is more widely applied across medical specialties. Some specialties require training in POCUS, and this training should begin in undergraduate medical education 1. Several professional societies advocate for the addition of ultrasound into undergraduate medical coursework, including the American Academy of Emergency Medicine, among others 1, 2. Early exposure to ultrasound has many potential benefits, including improved understanding of anatomy, physiology, pathology, and the physical exam 3.

An increasing number of medical schools are incorporating ultrasound into their curricula 1. At Texas College of Osteopathic Medicine (TCOM), students are exposed to ultrasound beginning in Year One. Training sessions are led primarily by Year Two students designated as ultrasound teaching assistants (TAs) via the near peer teaching model. The purpose of our study is to look at the effectiveness of near peer teaching in the setting of POCUS education. Near peer teaching occurs when material is taught to students by their peers. This has been proven to be an effective teaching technique in other settings and its utility in ultrasound continues to be explored 4, 5, 6. With each new class, POCUS education at TCOM has grown both in exposure time and clinical application. Ultrasound is implemented in the first year as part of the physical exam course and in the second year as part of the simulation lab course, both to correlate with the anatomy and physiology course material. To help facilitate this learning, POCUS TAs were introduced in 2019. The program was born out of necessity, as there was a limited number of POCUS-trained faculty available. Initially, sessions were taught by one faculty member using one POCUS device to large groups of students at a time. After recognizing that students had very little hands-on training with this teaching technique, near peer training was discussed. This allowed for smaller group sessions, even with few POCUS-trained faculty. This is a common problem faced by institutions when adding POCUS to the curriculum 3. In a recent survey of medical schools with ultrasound programs, 68.4% had 10 or fewer faculty involved with ultrasound, and most participated as volunteers 1. 

At TCOM, the TA program started with about 10 students from the class of 2022 who volunteered to help with student instruction. As more POCUS was added to the curriculum, student interest also grew. At the time of our project, the program had close to 30 TAs who were responsible for about 4 hours of instruction per week. After a year of implementation, curriculum staff decided to evaluate the efficacy of POCUS TAs and how near peer teaching may benefit both student teachers and learners. 

## Methods

Teaching assistants are second-year TCOM students who volunteered to lead the instruction of their peers. For the class of 2022 and 2023, TAs were selected informally and trained through both online modules (Sonosim™, Santa Monica, CA) and additional teaching sessions. The TAs then lead small group POCUS instruction during the physical exam or simulation courses under the supervision of POCUS faculty. Teaching assistants were responsible for approximately four hours of simulation lab teaching time each week. Prior to each new POCUS topic, students participated in a one-hour training session with faculty. Additional training time was not required but encouraged. While the TAs completed the majority of instruction, some students were occasionally placed in small groups led by faculty.

There was no risk associated with participating and students were consented to being part of the study prior to filling out the survey. To evaluate the effectiveness of TAs, TCOM students were surveyed on their experiences with the near peer teaching technique. At the time the survey was sent, both classes had experienced at least one year of near peer education. The class of 2022 was participating in clinical rotations and the class of 2023 was starting their second year of near peer teaching. Students were sent a survey that was fourteen questions in length. They were presented with a variety of phrases about their POCUS training and asked to rank their agreement with each phrase. The available options were strongly disagree, disagree, neutral, agree and strongly agree, or not likely, somewhat likely and very likely. Students who were identified as POCUS TAs were asked to complete a separate survey, in which they were asked about their experiences as both teachers and learners. The TA survey utilized a similar format previously mentioned and was fifteen questions in length. The surveys were limited to one response per student. Data from both surveys was collected and stored anonymously using an electronic survey system (Qualtrics™, Seattle, WA). This project was reviewed and approved through the North Texas Regional Institutional Review Board. 

## Results

For the general student population of approximately four-hundred students, sixty-three students respond to the survey, for a response rate of about 15.75%. We learned that 90.4% of these students either agreed or strongly agreed that POCUS is an integral part of medical education, and 79.4% of the students either agreed or strongly agreed that POCUS improves their understanding of systems-based course material. When looking at near peer preference versus other teaching methods, we found that 53.9% of the students prefer near peer techniques, while only 38.7% of the students would prefer faculty-led sessions. In terms of the effectiveness of this teaching style, 73% of the students agreed that their POCUS skills have improved with peer-led sessions. Finally, when assessing the lasting impact of POCUS education, 71.4% of the students agreed that peer-led sessions have made them want to pursue additional POCUS training, and 96.8% of the students reported that they are very likely or somewhat likely to POCUS in their future practice (Table 1). 

**Table 1:** General Student Responses to the Near Peer Ultrasound Evaluation Survey.

**General Student Population**					
**Survey Question**	**Strongly Disagree**	**Disagree**	**Neutral**	**Agree**	**Strongly Agree**
Ultrasound training has improved my understanding for system based courses.	0%	4.8%	15.9%	23.8%	55.6%
Ultrasound training is an integral part of medical education.	0%	3.2%	6.3%	33.3%	57.1%
I prefer near peer teaching techniques over other teaching techniques.	1.6%	12.7%	31.7%	28.6%	25.4%
I effectively learn during ultrasound peer taught sessions.	1.6%	9.5%	25.4%	44.4%	19.0%
I believe the ultrasound TAs are effective teachers and I have confidence in their skills.	1.6%	9.5%	23.8%	44.4%	20.6%
I would prefer faculty soley taught ultrasound skills.	4.8%	24.2%	32.3%	25.8%	12.9%
I feel my ultrasound skills have improved with near peer ultrasound training.	0%	6.3%	20.8%	49.2%	23.8%
I feel competent in my point of care ultrasound skills.	7.9%	22.2%	31.7%	25.4%	12.7%
Near peer ultrasound training has made me want to pursue more ultrasound training.	1.6%	6.3%	20.6%	44.4%	27.0%
**Survey Question**	**Not likely**	**Somewhat likely**		**Very Likely**	
How likely are you to pursue a residency with an ultrasound emphasis?	19.4%	53.2%		27.4%	
How likely are you to use point of care ultrasound in your future?	3.2%	57.1%		39.7%	

Among the TA group of forty-one students, nineteen students responded to the survey, for a response rate of 46.3%. Of the respondents, 78.9% had assisted with more than four teaching sessions, 84.2% attended more than four TA training sessions, and 94.7% of students also reported spending additional time practicing POCUS outside of TA activities each week. When looking at how being an POCUS TA impacted the students, we found that 100% of them either agreed or strongly agreed that being a TA has helped their medical education. Of the TAs, 78.9% either agreed or strongly agreed that they feel competent in their POCUS skills. When comparing different education methods, 78.9% of the TAs prefer near peer techniques over other teaching methods. Ultimately, we can conclude that being an POCUS TA had a large impact on the students, where 100% of TAs agreed or strongly agreed that being a TA has helped develop their POCUS skills and 100% of TAs are likely or very likely to use point of care POCUS in their future practice (Table 2). 

**Table 2:** Teaching Assistant Responses to the Near Peer Ultrasound Evaluation Survey.

**Teaching Assistant Population Survey**					
**Survey Question**	**Class of 2022**		**Class of 2023**		
Select your current year of training.	21.1%		78.9%		
**Survey Question**	**1**	**2**	**3**	**4**	**More than 4**
How many 2-hour simulation lab sessions have you helped with?	0%	5.3%	5.3%	10.5%	78.9%
How many practice sessions have you attended?	0%	5.3%	5.3%	5.3%	84.2%
**Survey Question**	**0 hours**	**Up to 1 hour**	**1-2 Hours**	**3 or more hours**	
On average, how much additional time weekly do you spend practicing ultrasound outside of TA activities?	5.3%	57.9%	36.8%	0%	
**Survey Question**	**Strongly Disagree**	**Disagree**	**Neutral**	**Agree**	**Strongly Agree**
Being an ultrasound TA has helped with my medical education.	0%	0%	0%	5.3%	94.7%
Being an ultrasound TA has hindered my medical education.	0%	0%	0%	26.3%	73.7%
I feel competent in my point of care ultrasound skills.	0%	5.3%	15.8%	52.6%	26.3%
I would recommend others to be an ultrasound TA.	0%	0%	0%	21.1%	78.9%
I prefer near peer teaching techniques over other techniques.	0%	0%	21.2%	47.4%	31.6%
Ultrasound TA training sessions prepare me to teach other students.	0%	0%	0%	42.1%	57.9%
Being an ultrasound TA has helped to develop my teaching skills overall.	0%	0%	0%	21.1%	78.9%
**Survey Question**	**Not Likely**	**Somewhat Likely**		**Very Likely**	
How likely are you to pursue a residency with an ultrasound emphasis?	5.3%	52.6%		42.1%	
How likely are you to use point of care ultrasound in your future practice?	0%	26.3%		73.7%	
How likely are you to pursue a career in which teaching is part of your clinical responsibilities?	5.3%	42.1%		52.6%	

## Discussion

There are many potential benefits to the integration of POCUS into undergraduate medical education 3. As reflected in our survey results, medical students feel that POCUS enhances their learning of traditional systems-based courses. When looking at how ultrasound is taught to medical students, there is a wide range of methods and approaches 1, 3. Based on our data, we propose that near peer instruction is a valuable method to teach POCUS for several reasons. First, when taught by their peers, students succeed in learning POCUS. 73% of our students agreed that their skills have improved through peer-led sessions. Teaching assistants improve their skills, as well; 78.9% of them agreed or strongly agreed that they are competent in POCUS. This success may be due, in part, to the fact that students explain concepts at a level that their peers can easily understand, as discussed in Naeger et al 5. Not only is near peer teaching effective, but students enjoy being taught by their classmates. We have found that a larger number of student respondents prefer peer-led POCUS sessions compared to other, more traditional teaching methods. Reasons for this may include better relationships with their colleagues and the low-stress environment of being taught by other students 3. Learners may feel more inclined to ask questions or participate when being instructed by someone who is academically their equal 5. Not only do these factors make the experience more enjoyable, but more conducive to learning. Our results also suggest that near peer instruction helps foster an interest in POCUS amongst students, where 96.8% of the students indicate that they are somewhat or very likely to use POCUS in their future practice. As mentioned previously, students may be more apt to participate in hands-on learning in front of their peers, which could lead to a more positive experience overall with POCUS. 

 Lastly, we believe that students who participate in our program as TAs have an even greater learning experience. 100% of them agreed or strongly agreed that being a TA has helped improve their POCUS abilities. Not only do they participate in POCUS as a learner, but they have the opportunity to develop their teaching skills, which will inevitably be used throughout their careers in medicine 3. In previous studies on peer-led teaching, student teachers reported improvements in their clinical, communication, and teaching skills 6. 

This study had several limitations, including the low response rate for the general student population and the fact that it was conducted at a single site. With a response rate of only 15.75%, there is the possibility of selection bias, as only the students with strong opinions on POCUS may have responded to the survey. There is a similar risk of bias with the TAs. Since TAs are volunteers, many of them had significant interest in POCUS beforehand and thus their positive survey results may reflect that. Other considerations include the challenge of standardizing TA skills. Despite training sessions, we recognize that there may be discrepancies in TA ability, which may impact the student experience. This issue was highlighted in Smith et al, in which some of the student teachers had varying levels of preparedness and confidence 6. Furthermore, many of our survey questions were subjective. For example, we did not formally assess whether students improved with near peer instruction. Lastly, there may be variations in the amount of exposure to other, different teaching modalities. While the class of 2023 has experienced other teaching techniques throughout their time at TCOM, they only briefly were exposed to purely faculty-led POCUS instruction. This is because the TA program was already established by the time they entered medical school. Strengths to the study include the anonymity of the surveys, as it allowed for students to share their thoughts without concern for repercussions. Similarly, the surveys were easily accessible via email and could be completed on any device. We must also consider that the positive response to the near peer model may be a reflection of the TCOM student population. A large portion of students at TCOM tend to be driven towards specialties such as primary care, internal medicine, and emergency medicine, where POCUS is of growing interest. 

The results of our surveys not only have implications for medical education, but for clinical medicine, as well. Many of TCOM’s students plan to train and practice in rural or underserved areas. The skills they have learned through our POCUS program will help them bring POCUS to their communities and beyond. With added teaching experience, our TAs are prepared to instruct fellow physicians, advanced practice providers, and other members of their community in POCUS. This demonstrates how the near peer model of teaching is incredibly sustainable. As students gain more experience, they themselves become teachers and can disseminate information and skills to others. 

Moving forward, the POCUS leadership has developed an evaluation process to standardize TA skills and further standardize the POCUS experience at TCOM. When discussing standardization, a question that was presented was how to quantify and qualify POCUS as a learned skill. Currently in the development and trial phase is a credentialing program to train and test third and fourth-year medical students on their POCUS abilities. Teaching assistants will be an integral part of this program, as students, providers, and other community members earn certifications in POCUS. We also hope this program will provide another opportunity to learn about how POCUS can best be integrated into medical education

## Conclusion and Future Directions

Our study has shown that it is possible to develop and implement a near peer POCUS curriculum that is well received and beneficial for students. To further expand this study, it would be valuable to survey how other medical schools have implemented POCUS education and how it was received. Future directions also include obtaining feedback on TA abilities from practicing physicians. 

## Disclosures

None.
